# Supplementary material for: The Structure and Diversity of Nitrogen Functional Groups from Different Cropping Systems in Yellow River Delta
Source: Microorganisms. 2020 Mar 17;8(3):424. doi: 10.3390/microorganisms8030424 (PMC7144021; doi:10.3390/microorganisms8030424)
Supplement: Supplementary file 1 [file microorganisms-08-00424-s001.pdf]

Supplementary Table 1. The description of sites in this study, including fertilization, density and height of growing plants

Cropping systems		Fertilization	Coverage (%) in 1x1m^2	Height (m)
Wheat-Corn (*Triticum aestivum* L.-*Zea mays* L.)		Compound fertilizer 600 kg h^{-1}year^{-1}+ diammonium hydrogen phosphate 550 kg h^{-1}year^{-1}	30-50	0.15-0.2
Cotton (*Gossypium hirsutum* L.)		Compound fertilizer 300 kg h^{-1}year^{-1}	70-80	0.5-0.7
Rice (*Oryza sativa* L.)		Ammonium sulphate 280 kg h^{-1}year^{-1}+ Potassium chloride100 kg h^{-1}year^{-1}	30-40	0.3-0.4
Soybean-Corn (*Glycine max* L.- *Zea mays* L.)		Compound fertilizer 280 kg h^{-1}year^{-1}	70-80	0.5-0.7
Fruit-Vegetable	Strawberry (*Fragaria* × *ananassa* Duch.)	Compound fertilizer 280 kg h^{-1}year^{-1}	70-80	0.15-0.22
	Pepper (*Capsicum annuum L.*)		60-70	0.4-0.55

Supplementary Table 2. Network properties of *amoA*-AOA and *nirS*-type denitrifier communities.

	cropping system	clustering coefficient	connected components	network diameter	network centralization	characteristic path length	number of nodes	number of edges	network density
amoA -AOA	Wheat-Corn	0.669	4	11	0.171	3.722	44	132	0.14
	Cotton	0.728	4	7	0.149	2.572	46	128	0.124
	Rice	0.7	4	5	0.12	1.748	29	56	0.07
	Soybean-Corn	0.613	8	4	0.07	1.204	31	47	0.101
	Fruit	0.527	5	5	0.077	2.105	38	63	0.09
nirS-type denitrifier	Wheat-Corn	0.748	6	3	0.088	1.26	35	73	0.163
	Cotton	0.781	5	4	0.125	1.419	34	70	0.125
	Rice	0.774	4	4	0.157	1.634	22	44	0.19
	Soybean-Corn	0.8	6	1	0.123	1	20	29	0.123
	Fruit	0.952	4	2	0.068	1.065	33	115	0.128
